# Revelation of mRNAs and proteins in porcine milk exosomes by transcriptomic and proteomic analysis

**DOI:** 10.1186/s12917-017-1021-8

**Published:** 2017-04-13

**Authors:** Ting Chen, Qian-Yun Xi, Jia-Jie Sun, Rui-Song Ye, Xiao Cheng, Rui-Ping Sun, Song-Bo Wang, Gang Shu, Li-Na Wang, Xiao-Tong Zhu, Qing-Yan Jiang, Yong-Liang Zhang

**Affiliations:** 1 National Engineering Research Center For Breeding Swine Industry, Guandong Provincial Key Laboratory of Agro-Animal Genomics and Molecular Breeding, Guandong Province Research Center of Woody Forage Engineering and Technology, South China Agricultural University, 483 Wushan Road, Guangzhou, 510642 China; 2 Institute of Animal Science and Veterinary Medicine, Hainan Academy of Agricultural Sciences, Haikou, 571100 China

**Keywords:** Porcine milk exosomes, RNA-seq, Proteomic analysis

## Abstract

**Background:**

Milk is a complex liquid that provides nutrition to newborns. Recent reports demonstrated that milk is enriched in maternal-derived exosomes that are involved in fetal physiological and pathological conditions by transmission of exosomal mRNAs, miRNAs and proteins. Until now, there is no such research relevant to exosomal mRNAs and proteins in porcine milk, therefore, we have attempted to investigate porcine milk exosomal mRNAs and proteins using RNA-sequencing and proteomic analysis.

**Results:**

A total of 16,304 (13,895 known and 2,﻿409 novel mRNAs) mRNAs and 639 (571 known, 66 candidate and 2 putative proteins) proteins were identified. GO and KEGG annotation indicated that most proteins were located in the cytoplasm and participated in many immunity and disease-related pathways, and some mRNAs were closely related to metabolisms, degradation and signaling pathways. Interestingly, 19 categories of proteins were tissue-specific and detected in placenta, liver, milk, plasma and mammary. COG analysis divided the identified mRNAs and proteins into 6 and 23 categories, respectively, 18 mRNAs and 10 proteins appeared to be involved in cell cycle control, cell division and chromosome partitioning. Additionally, 14 selected mRNAs were identified by qPCR, meanwhile, 10 proteins related to immunity and cell proliferation were detected by Western blot.

**Conclusions:**

These results provide the first insight into porcine milk exosomal mRNA and proteins, and will facilitate further research into the physiological significance of milk exosomes for infants.

**Electronic supplementary material:**

The online version of this article (doi:10.1186/s12917-017-1021-8) contains supplementary material, which is available to authorized users.

## Background

Milk is the primary source of nutrition for newborns, and breastfeeding is known to make a valuable contribution to infant health [1]. Breast milk contains a potent mixture of diverse components including milk fat globules (MFG), immune competent cells, antibodies, soluble proteins, cytokines, and antimicrobial peptides [2] that together protect young infants against infections [3]. In addition, the milk contains growth factors which could promote intestinal development [4] and may protect infants against developing allergies [5]. Meanwhile, milk also contain many microvesicles, such as milk-derieved exosomes, who was reported to transfer contained RNAs to living cells and influenced the development of calf’s gastrointestinal and immune systems [6].

Exosomes are small membrane vesicles (30–100 nm) which released from producing cells into the extracellular environment [7]. Many different cell types have the capacity to produce and release exosomes [8–13]. Additionally, milk-derived exosomes have been reported in humans, cows and pigs [14–17] and which involved in many biological processes. Exosomes contain proteins, mRNAs, miRNAs and lipids. Recent studies revealed that human [18], bovine [19], pig [20], and rat [21] milk contain miRNAs, and mRNAs have also been identified in whey [6, 21–23]. 10,948 mRNA transcripts were detected in rat milk, and some immune and development-related mRNAs showed time-dependent expression [21]. 19,320 mRNAs were detected by microarray analyses in bovine milk exosomes, and they had possible effects of human cells [24]. Additionally, Cecilia Lässer et al. demonstrated that mRNAs in breast milk exosomes could be taken up by human macrophages [25].Until now, the components of mRNAs in porcine milk exosmes are still unclear.

Proteins in exosome were dependented on the specific cell-type [26], the dendritic cell-derived exosomes contain several cytosolic proteins [8]. Body fluid derived exosomes CD24, CD9, Annexin-1 and Hsp70 were as positive marker proteins [27]. Anti-MHC-class II- and anti-CD63 beads were used to isolate human breast milk exosmes [28]. In bovine milk exosomes 2,107 proteins were identified, and all major exosome protein markers were abundant [29], as were milk fat globule membrane (MFGM) proteins. Another report showed 2,350 proteins in bovine milk exosome via iTRAQ, and 90 exosomal proteins were found to be differentially regulated by infections [30].

In our previous study, miRNAs in porcine milk exosomes have been revealed by deep sequencing [17], but up to now, porcine milk exosomal mRNAs and proteins remains unknown. Therefore, we further performed RNA-sequencing and proteomic analysis of porcine milk exosomes in order to understand new physiological functions, especially immunity and proliferation related regulation of porcine milk.

## Methods

### Milk sample preparation

Fresh porcine milk samples were collected from 10 healthy Landrace female pigs that had been lactating for 1 to 5 days (after parturition) at the pig farm of the South China Agriculture University (Guangzhou, China). Milk samples were frozen immediately and kept at −80 °C until used.

### Isolation of milk exosomes

Porcine milk exosomes were separated as previously described [17]. Briefly, about 80–100 mL fresh raw porcine milk samples were centrifuged at 2,000 g for 30 min at 4 °C to remove milk fat globules (MFGs) and mammary gland-derived cells [18]. Defatted samples were then subjected to centrifugation at 12,000 g for 30 min at 4 °C to remove residual MFGs, casein, and other debris [6]. From the supernatant, the membrane fraction was prepared by ultracentrifugation at 110,000 g for 2 h using an SW41T rotor (Beckman Coulter Instruments, Fullerton, CA). Then, the exosome purification steps were as previously described [29, 30].

### RNA isolation

Total RNA was isolated from porcine milk exosome samples by Trizol reagent (Invitrogen, Carlsbad, CA) according to the manufacturer’s protocol. The quality of RNA was examined by 2% agarose gel electrophoresis and with a BiophotometerNanoDrop 2000 (Thermo, USA), as well as further confirmed using a Bioanalyzer (Agilent Technologies, Santa Clara, CA).

### RNA-sequencing

The collected RNA samples were analyzed by IlluminaHiSeq™ 2000 analyzer at Beijing Genomics Institute(BGI, Shenzhen, China) as previously described [31]. Firstly, poly (A) mRNA was isolated from total RNA sample with Oligo(dT) magnetic beads. Secondly, the purified mRNA was fragmented by the RNA fragmentation kit (Ambion), the first-strand cDNA synthesis was performed using random hexamer primers and reverse transcriptase, and the second-strand cDNA was synthesized using RNase H and DNA polymerase I. Then the cDNA libraries were prepared using the Illumina Genomic DNA Sample Prep kit (Illumina) following the manufacturer’s protocol. Finally, the library was sequencing using Illumina HiSeq™ 2000.

### Sequencing analysis

The porcine reference genome sequence and annotated transcript set were downloaded from the ensemble database (Sscrofa10.2, http://asia.ensembl.org/Sus_scrofa/Info/Index). After quality control (QC) step of raw reads, then removing low quality reads, reads containing Ns > 5 and reads containing adapters, clean reads were aligned to the reference pig genomic database (Sscrofa 10.2,) with SOAPaligner/SOAP2 [32] and allowing up to 5 mismatches in 90-bp reads. The alignment data were utilized to calculate distribution of reads on pig gene database (http://www.ncbi.nlm.nih.gov/), and the numbers of reads per kilobase of dexon region in a gene per million mapped reads were used as the value of normalized gene expression levels [33]. The unalignment data carried out novel transcript prediction, reads are at least 200 bp away from annotated gene, the transcript is of length over180 bp and the sequencing depth is no less than 2 for novel transcript unit analysis.

### qPCR identification of known mRNAs in porcine milk exosome

Total RNA (identical with the RNA-sequencing sample) was first digested with DNase I (Promega, American), and 2 μg of total RNA was reverse transcribed by oligo (dT).The cDNA was diluted by 2-fold with ddH_2_O, and PCR was performed on a Bio-Rad system (BIO-RAD, USA) in a final 20 μL volume reaction, containing 2 μL PCR cDNA, 10 μL of 2× PCR Mix (Roche, Switzerland) and 1 mM of each primer. The real-time PCR thermal profile was as follows: 5 min at 95 °C, 40 cycles of 30 s at 94 °C, 30 s at the corresponding annealing temperature (Tm) and 72 °C for 30 s, followed by 72 °C at 10 min, and 5S ribosomal RNA was used as an internal control for the PCR [17, 34]. The mRNAs primers were designed with Primer 5.0 (Table 1).

**Table 1:** Primers for qPCR

ID	Primer	Sequences (5’to3’)	Products length (bp)
ENSSSCG00000000207	LOC100739053-F	CAAAGGAAGCCTACAAGAA	198
	LOC100739053-R	CACGGTAGTCCAGCAGA	
ENSSSCG00000003930	RPS8-F	GAGAAAGCCCTACCACA	191
	RPS8-R	CGTCAATAATCCTCGTCT	
ENSSSCG00000004489	EF1ALPH-F	GATTGTTGCTGCTGGTGT	226
	EF1ALPH-R	TGCTACTGTATCAGGGTTGT	
ENSSSCG00000004177	RPS12-F	TCTACCCGTAACCCACC	219
	RPS12-R	CCTCCACCAACTTGACATA	
ENSSSCG00000015103	RPS25-F	GCCCAAGGACGACAA	109
	RPS25-R	GCCTTTGGACCACTTC	
ENSSSCG00000006249	RPS20-F	ACCGCTGTTCGCTCTTC	211
	RPS20-R	GTCCCTTCACTTTGAGGTTCT	
ENSSSCG00000029830	RPL8-F	CGAGCGACACGGCTACAT	255
	RPL8-R	GGCTTCTCCTCCAGACAACAC	
ENSSSCG00000009267	CSN3-F	CACCTGAGACCACCACT	140
	CSN3-R	TGACTGAAGGCAGATAA	
ENSSSCG00000013907	UBA52-F	ACGGGCAAGACCATCAC	196
	UBA52-R	GCAGACGAAGCACCAAGT	
ENSSSCG00000001502	RPS18-F	AGGGTGTAGGACGGAGAT	134
	RPS18-R	CTTGTATTGGCGAGGATT	
ENSSSCG00000024825	RPL6-F	CAGAGGCAAGAGGGTCA	128
	RPL6-R	TGGTGGAGGTGGCAATA	
ENSSSCG00000004970	RPLP1-F	GCACGACGATGAGGTTAC	131
	RPLP1-R	TGAGGCTCCCGATGTT	
ENSSSCG00000010328	RPS24-F	TTGATGTCCTTCACCCTG	269
	RPS24-R	CATTCTGTTCTTGCGTTCT	
ENSSSCG00000025527	FABP3-F	GCTGGGATTGAAGTTTGA	163
	FABP3-R	GTGGGTGAGTGTCAGGATG	
5S	5S–F	TCTACGGCCATACCACCCTGAA	83
	5S–R	GGCCCGACCCTGCTTAG	

### Total protein extraction

RIPA lysis buffer was used to extract porcine milk exosomal proteins according to the assay kit protocol (Bioteke, Beijing). Briefly, 1 mM PMSF was added to the RIPA lysis buffer and 100–200 μL was added to porcine milk exosomes. Following complete exosome lysis, the sample was centrifuged at 10,000–14,000 g for 3–5 min and the supernatant was subjected to further analysis. Proteins were stored at −80 °C until used.

### Protein separation by 1D SDS-PAGE and in-gel digestion

Porcine milk exosome proteins were resolved by 12% polyacrylamide gel. The gel was stained with Coomassie blue R-250. 20 bands were excised and destained using 50 mM ammonium bicarbonate in 50% ACN. And then the gel pieces were performed incubating with 10 mM DTT in 25 mM ammonium bicarbonate for 1 h at 60 °C to reduce disulfide bonds and incubating the samples with 55 mM iodoacetamide in 25 mM ammonium bicarbonate for 45 min at room temperature in dark for Alkylation of cysteines. Then, using the Trypsin Gold (Promega, Madison, WI, USA) for digested (37 °C, 16 h) the gel bands. After the peptides sequentially extracted from gel bands by 0.1% formic acid in 50% ACN twice, using 100% ACN twice, the extracted peptides were dried and stored at −80 °C until LC-MS/MS analysis.

### Protein sequencing

Protein samples were analyzed using a Q-EXACTIVE mass spectrometer at the Beijing Genomics Institute (BGI, Shenzhen, China). Briefly, samples were separated by 1D SDS-PAGE and in-gel digestion was performed to generate peptides for LC-MS/MS analysis. Peptide fractions were initially separated on a LC-20 AD nanoHPLC (Shimadzu, Kyoto, Japan), then subjected to nanoelectrospray ionization followed by tandem mass spectrometry (MS/MS) using a Q EXACTIVE (ThermoFisher Scientific, San Jose, CA) coupled online to the HPLC.

### LC-ESI-MS/MS analysis based on Q EXACTIVE

After a series of processing, we regulated each fraction at the average final concentration of peptide at 0.5 μg/uL and loading 10 uL on a LC-20 AD nanoHPLC (Shimadzu, Kyoto, Japan) by the autosampler onto a 2 cm C18 trap column. Then 10 cm analytical C18 column (inner diameter 75 μm) was used for eluted the peptides. After the sample was loading to the trap column, then bring into the analytical column, and finally the separated peptides were subjected to nanoelectrospray ionization followed by tandem mass spectrometry (MS/MS) in a Q EXACTIVE (ThermoFisher Scientific, San Jose, CA) coupled online to the HPLC. Resolution of 7,000 on Orbitrap was used to detect the intact peptides. Peptides were selected for MS/MS using high-energy collision dissociation (HCD) operating mode with a normalized collision energy setting of 27.0; ion fragments were setting of a resolution of 17,500. A data-dependent procedure that alternated between one MS scan followed by 15 MS/MS scans was applied for the 15 most abundant precursor ions above a threshold ion count of 20,000 in the MS survey scan with a following Dynamic Exclusion duration of 15 s. The electrospray voltage applied was 1.6 kV. The Automatic gain control (AGC) which used to optimize the spectra generated by the orbitrap was target for full MS was 3e6 and 1e5 for MS2. For MS scans, the m/z scan range was 350 to 2,000 Da. For MS2 scans, the m/z scan range was 100–1,800. All those works were carried out in Beijing Genomics Institute (BGI, Shenzhen, China).

### Protein data analysis

All raw data were acquired using an Orbitrap, converted to MGF files using Proteome Discoverer 1.2 (PD1.2, Thermo), and the Mascot search engine (Matrix Science, London, UK; version 2.3.02) was used to search against a database containing 25,152 sequences(ftp://ftp.ensembl.org/pub/release-73/fasta/sus_scrofa/pep/).Non-intact (>20 ppm) peptides and fragmented ions (0.6 Da) were removed, with allowance for one missed cleavage in trypsin digests. Next, the fixed carbamidomethyl (C) modification, and potential variable modifications Gln- > pyro-Glu (N-term Q), oxidation (M), deamidation (NQ), and +2 and +3 charge states were considered. Mascot was used to search the automatic decoy database by choosing the decoy checkbox, with the decoy checkbox set to generate a random sequence of database and test for raw spectra, as well as the actual database. Finally, only peptides with significance scores ≥20 at the 99% confidence interval in the Mascot probability analysis were counted as identified proteins [29]. All identified proteins included at least one unique peptide.

### Western blot identification

Protein samples (20–30 μg) were measured by BCA assay [35], and separated using 10–15% SDS-PAGE, transferred to a 0.22 μm or 0.45 μm polyvinylidenedifluoride membrane (Millipore), and incubated with specific and HRP-conjugated secondary antibodies, and detected with an enhanced chemiluminescence kit (Roche, Switzerland) using FluorChem M (Proteinsimple) [36]. Anti-EGF (AB20578b), anti-TGFB-3 (AB20578b), anti-MSTN (AB60418a), connective tissue growth factor (CTGF) (AB60212a), anti-PDGFA (AB61078b), anti-CD63 (D260973), anti-IGFBP-7 (AB60509b), anti-CD9 (AB54118), anti-HTRA3 (AB61337a), and anti-THBS1 (AB61391a) were purchased from BBI Antibody (SangonBiotch, Shanghai, China). Lactoferrin (C-15) and β-actin were purchased from Santa Cruz (Santa Cruz, American). Protein’ concentrations were determined using the Pierce BCA Protein Assay Kit (Thermo Fisher, American) using a BSA standard.

### Bioinformatics analysis

We performed functional annotation using Blast2GO to search the non-redundant protein database (NR; NCBI) and the COG database (http://www.ncbi.nlm.nih.gov/COG/), which was used to classify and group the identified proteins. All the known mRNAs and proteins were performed Gene Ontology, KEGG pathway and Tissue-specific using DAVID6.7 bioinformatics resources (http://david.abcc.ncifcrf.gov/).

## Results

### Identification of exosomes by western blotting and extraction of RNA and protein from porcine milk exosome

**Fig. 1:**
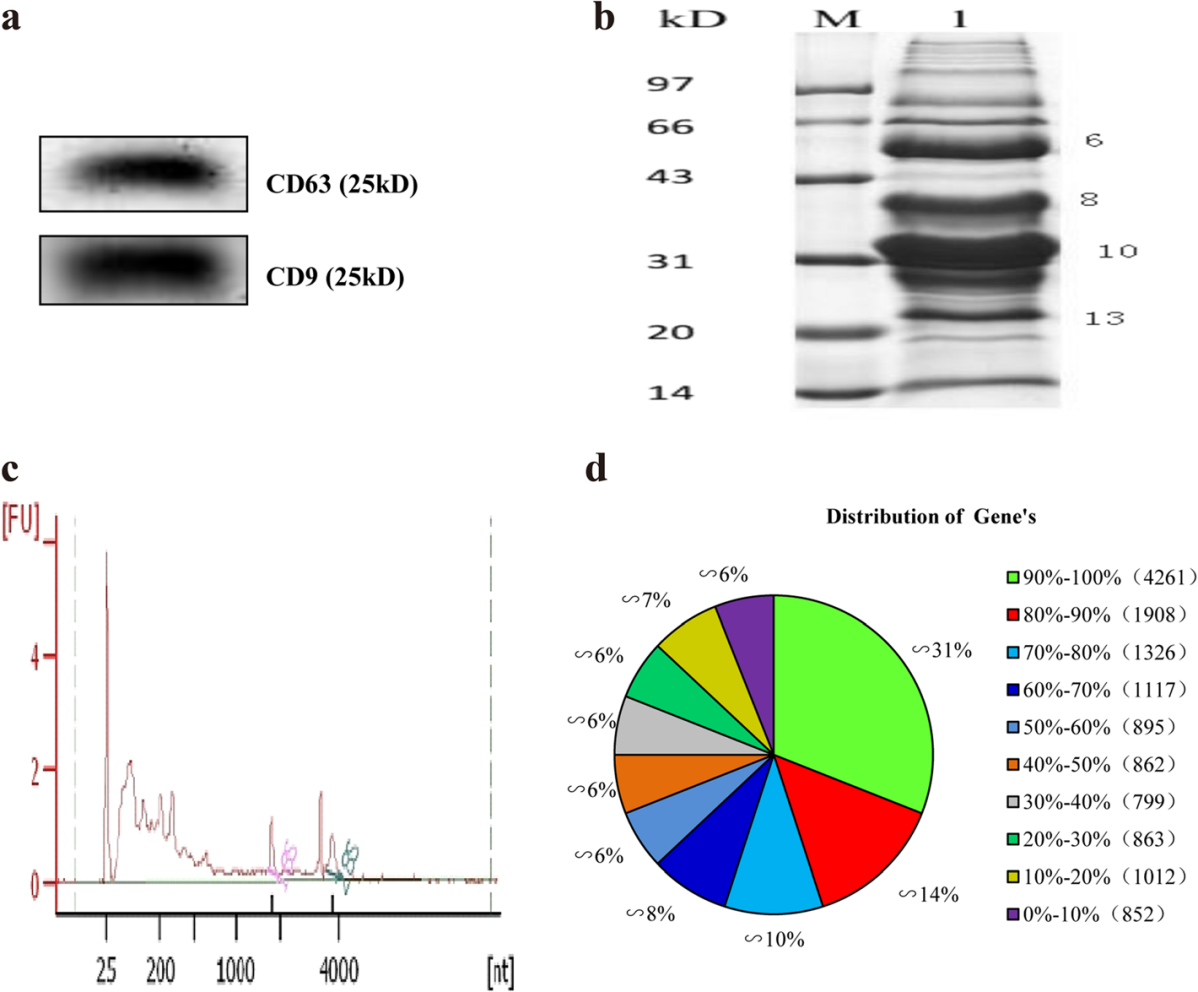
Identification of proteins and mRNAs in porcine milk exosomes. **a** detection of the exosomal marker proteins CD63 and CD9 by Western blotting. **b** SDS-PAGE. **c** RNA sample analyzed by the Agilent Bioanalyzer 2100. **d** distribution of genen’s coverage

We previously isolated exosomes from porcine milk and analyzed them using transmission electron microscopy [17]. In the present study, we observed exosomal marker proteins CD63 and CD9 by Western blotting (Fig. 1a). We extracted total RNA from the pellets after ultracentrifugation and examined the RNA by Agilent 2100, and the results showed that the porcine milk exosome contained RNAs and small rRNAs (Fig. 1c), which is consistent with previous studies [4, 6, 17, 20]. Porcine milk proteins were extracted using RIPA lysis buffer and resolved using SDS-PAGE (Fig. 1b), which proteins covered a large molecular weight range, but most of them were fell into the 20–25, 28–35, 35–40 and 43–55 kDa ranges, and these ranges were considered separately.

### Transcript sequencing and analysis

#### Transcript sequencing

We totally obtained 77,106,888 raw reads, which mapped to porcine genome (**sscrofa10.2,** www.ensembl.org/Sus_scrofa/). The mapped proportion was 63.76% accounting for 49,161,814 reads, and the perfect match reads were 33,863,808 (43.92%) and the unique match reads were 45,080,932 (58.47%). By blast searching the 77,106,888 reads against pig coding gene database (http://www.ncbi.nlm.nih.gov/), 57,413,016 total match reads (represented 74.46%) and 53,836,128 (69.82%) unique matched reads were identified (Table 2). All the reads represent 13,895 genes (Additional file 1), the subsequent distribution of genes’ coverage analysis showed the number of genes’ coverage >50% contained 9,507 genes and represented 69% of 13,895 genes, the 4,261 (representing 31%) genes coverage are 90%–100% (Fig. 1d).

**Table 2:** Alignment statistics of RNA-seq data map to reference genome and gene database

	Map to Genome		Map to Gene	
	Reads number	Percentage	Reads number(control)	Percentage
Total Reads	77,106,888	100.00%	77,106,888	100.00%
Total Base Pairs	6,939,619,920	100.00%	6,939,619,920	100.00%
Total Mapped Reads	49,161,814	63.76%	57,413,016	74.46%
perfect match	33,863,808	43.92%	45,400,239	58.88%
<=5 bp mismatch	15,298,006	19.84%	12,012,777	15.58%
unique match	45,080,932	58.47%	53,836,128	69.82%
multi-position match	4,080,882	5.29%	3,576,888	4.64%
Total Unmapped Reads	27,945,074	36.24%	19,693,872	25.54%

#### Novel mRNAs predicted in pig exosome milk

**Fig. 2:**
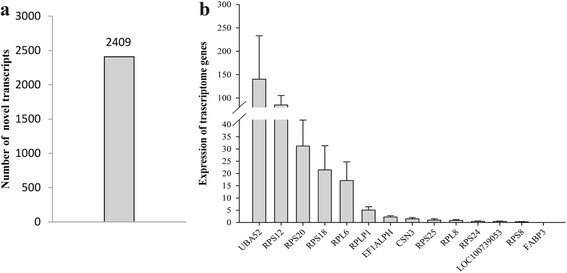
Statistics of novel transcripts and qPCR detected randomly from top 50 list in RNA-sequencing. **a** The number of predicted novel transcripts in porcine milk exosome. **b** The expression of 14 mRNAs, from left to right, respectively: UBA52, RPS12, RPS20, RPS18, RPL6, RPLP1, EF1ALPH, CSN3, RPS25, RPL8, RPS24, LOC100739053, RPS8, FABP3

Then we performed a novel transcript prediction and annotation according to the criteria described in Method. Results showed we obtained 2,409 novel transcripts (Fig. 2a and Additional file 2), and those novel transcripts were distributed in all the 19 chromosomes. These results would improve the gene annotations of the porcine genome and transcriptome [31].

#### qPCR identified for mRNAs

After a series of analysis of RNA sequencing, we randomly selected 14 transcripts genes from the top 50 list (Additional file 3) for evaluated their expression in the porcine milk exosomes by qPCR. The results showed that they were all detected in the sample (Fig. 2b).

#### Proteome sequencing and data analysis

Following separation by SDS-PAGE, in-gel digestion was performed and peptides were analyzed by mass spectroscopy. The four groups of P130340_6, P130340_8, P130340_10 and P130340_13 (6, 8, 10, and 13 in Fig. 1b) were corresponding to 43–55, 35–40, 28–35 and 20–25 kDa, respectively, which were treated identically, since they displayed a relatively high gray density in the gel. With a false discovery rate (FDR) setting ≤1.2%, 307,390 total spectras were detected, which only 18,638 spectras could be mapped using the Mascot software, and 2,313 peptides represent 639 proteins were ultimately identified from the sample (*Sus_scrofa*, Table 3 and Additional file 4), and which number of protein matched with a given quality match check criterion with at least possessing one unique peptide can be considered as a reliable protein. Of these, 571 proteins were present in the *Sscrofa* 10.2 database, 66 were novel candidate proteins and two were putative proteins (Additional file 4). Most of the novel proteins (44) and the two putative proteins were not highly abundant, whereas most of high abundance proteins were known proteins. Analysis of protein and peptide length distribution after digestion revealed that most were between 8 and 54 amino acids, and the majorities were between 9 and 25 residues, with the highest proportion (12%) comprising 13 amino acids (Additional file 5: Figure S1). Analysis of the peptide and spectrogram distribution showed that lots of proteins were represented by between 1 and 10 unique peptides, and one unique peptide was the predominantly case (Additional file 6: Figure S2). In the sequence coverage range of 0% to 20%, 473 proteins were identified (77.02%, Additional file 7: Figure S3e), and the sequence coverage was increased as the number of identified proteins decreased (Additional file 7: Figure S3a, b, c, d, e).

**Table 3:** Proteins identified in this study

Sample	Total spectra	Identified spectra	Identified peptides	Identified proteins	FDR (%)	Unknown protein	Putative protein
P130340_10	13,430	672 [5.0037%]	92	40	1.05	4	
P130340_13	17,281	790 [4.5715%]	108	44	0.90	2	
P130340_6	17,783	721 [4.0544%]	303	94	1.13	12	
P130340_8	15,140	596 [3.9366%]	180	59	1.03	4	
Sus_scrofa	307,390	18,638 [6.0633%]	2,313	639	1.04	66	2

#### Identification by Western blotting

**Fig. 3:**
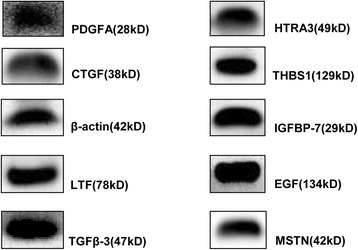
Confirmation by Western blotting. All 10 randomly selected proteins were confirmed to be present in porcine milk exosome

Based on the above results, we randomly selected 10 proteins to confirm their presence in porcine milk exosomes. Specifically, EGF, TGFβ-3, MSTN, CTGF, IGFBP-7, PDGFA, HTRA3, THBS1, β-actin and lactoferrin (LTF) were all successfully detected (Fig. 3).

#### COG annotation of mRNAs and proteins

**Fig. 4:**
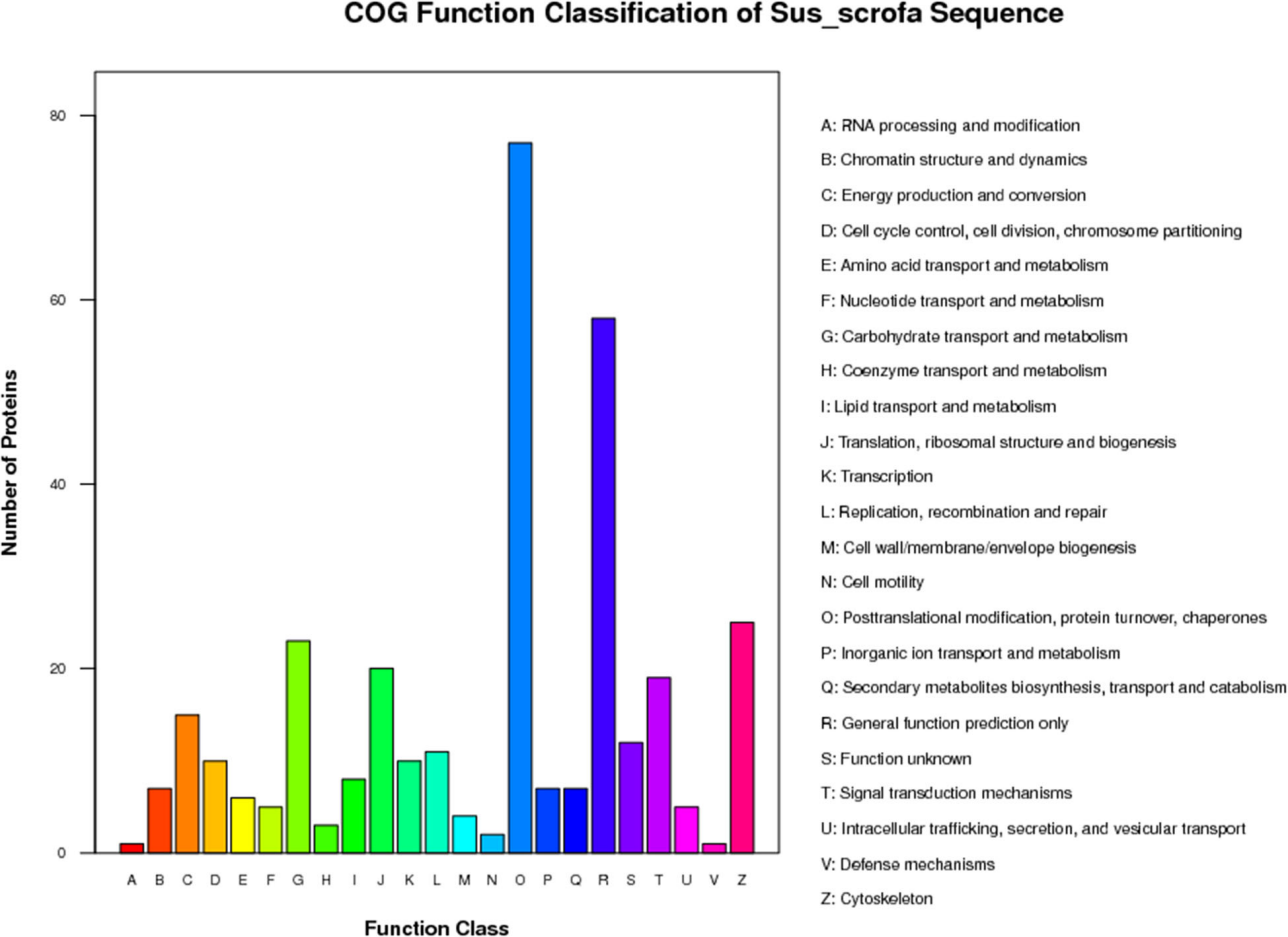
COG annotation of porcine milk exosome and Sus_Scrofa database proteins

**Fig. 5:**
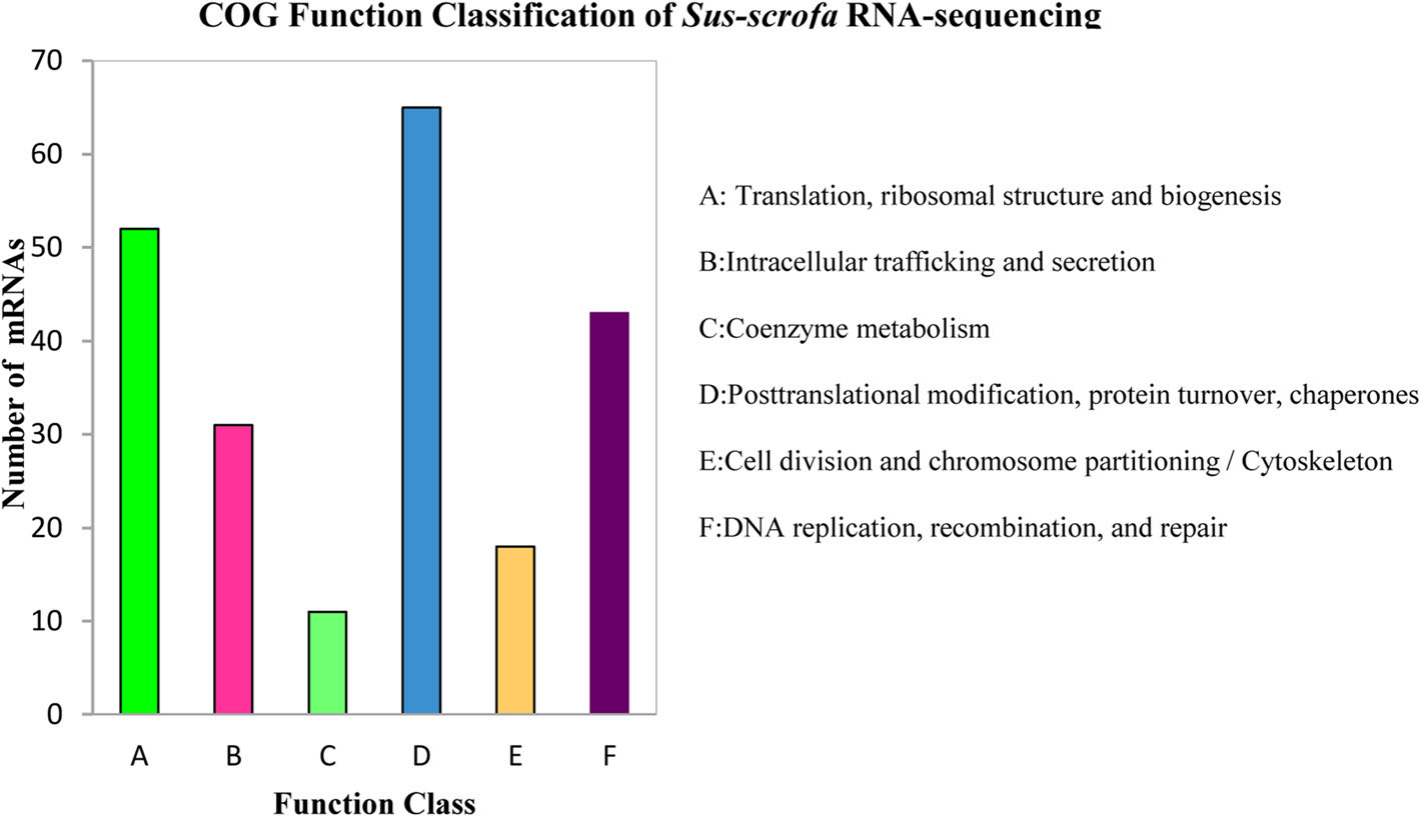
COG annotation of porcine milk exosome and Sus_Scrofa database mRNAs

The Cluster of Orthologous Groups of proteins (COG) database was used for protein orthologous classification, and all proteins in this database are assumed to be derived from a common protein ancestor. COG analysis showed that proteins from porcine milk exosomes were connected with multiple biological processes (Fig. 4 and Additional file 8). Interestingly, proteins involved in DNA or RNA synthesis and transport particularly abundant. Furthermore, five proteins were related to intracellular trafficking, secretion, and vesicular transport, with some in the high abundance P130340_13 (Additional file 9: Figure S4b) and P130340_8 groups (Additional file 9: Figure S4d). Additionally, 10 conserved proteins were involved in cell cycle control, cell division and chromosome partitioning. Similarly, enriched 6 COG Ontology in mRNAs, including 31 genes related to intracellular trafficking and secretion and 18 mRNAs of Cell division and chromosome partitioning / Cytoskeleton (Fig. 5 and Additional file 10).

#### Go analysis of mRNAs and proteins

GO annotation was performed using DAVID version 6.7 (http://david.abcc.ncifcrf.gov) with a standard Benjamini < 0.05. We selected the top 10 GO terms of Cellular Component (CC), Molecular Function (MF) and Biological Process (BP) for further analysis. For mRNA, cytoplasm genes account for a high proportion (6.3%), and specific intracellular organelle lumen, nuclear lumen genes account for ~1.9%. Predicted functions included various bindings (including adenyl ribonucleotide, magnesium ion, nuclear hormone receptor and protein kinase) and diverse enzymatic activity (including protein kinase, pyrophosphate, transcription coactivator, exonuclease, small conjugating protein ligase and NADH dehydrogenase), predicted biological processes relative to proteins (include protein metabolic, transport, modification and catabolic process) and RNA (including RNA metabolic, processing and ncRNA processing) (Table 4 and Additional file 10). For proteins, most of them were included in cytoplasm and cytoplasmic part, taking a proportion of 7.1%. Additionally, there were lots of specific membrane-bounded vesicle lumen, granule lumen, vesicle, lytic vacuole and reticulum lumen proteins. And major of those proteins were enriched in the molecular function in terms of diverse activity and predicted biological processes, including acute inflammatory response, complement activation, classical pathway, B cell mediated immunity, negative regulation of blood coagulation and coagulation, activation of immune response and protein maturation and processing (Table 5 and Additional file 5).

**Table 4:** GO annotation of identified mRNAs

Category	Term	Count	%	Benjamini
GOTERM_BP_5	GO:0044267 ~ cellular protein metabolic process	1,481	1.30	2.054E-42
GOTERM_BP_5	GO:0015031 ~ protein transport	531	0.47	3.262E-27
GOTERM_BP_5	GO:0016070 ~ RNA metabolic process	633	0.56	5.403E-27
GOTERM_BP_5	GO:0006396 ~ RNA processing	388	0.34	1.149E-21
GOTERM_BP_5	GO:0006464 ~ protein modification process	888	0.78	8.933E-18
GOTERM_BP_5	GO:0006886 ~ intracellular protein transport	271	0.24	1.149E-16
GOTERM_BP_5	GO:0030163 ~ protein catabolic process	404	0.36	3.782E-12
GOTERM_BP_5	GO:0043632 ~ modification-dependent macromolecule catabolic process	374	0.33	1.875E-11
GOTERM_BP_5	GO:0044257 ~ cellular protein catabolic process	390	0.34	2.34E-11
GOTERM_BP_5	GO:0034470 ~ ncRNA processing	141	0.12	4.795E-10
GOTERM_CC_5	GO:0005737 ~ cytoplasm	4,253	3.74	1.54E-126
GOTERM_CC_5	GO:0043231 ~ intracellular membrane-bounded organelle	4,485	3.94	5.61E-99
GOTERM_CC_5	GO:0044444 ~ cytoplasmic part	2,924	2.57	9.8E-86
GOTERM_CC_5	GO:0044446 ~ intracellular organelle part	2,530	2.22	5.426E-71
GOTERM_CC_5	GO:0070013 ~ intracellular organelle lumen	1,200	1.06	3.933E-66
GOTERM_CC_5	GO:0044428 ~ nuclear part	1,205	1.06	6.521E-59
GOTERM_CC_5	GO:0031981 ~ nuclear lumen	979	0.86	5.556E-53
GOTERM_CC_5	GO:0005829 ~ cytosol	879	0.77	2.958E-41
GOTERM_CC_5	GO:0005634 ~ nucleus	2,801	2.46	1.904E-33
GOTERM_CC_5	GO:0005654 ~ nucleoplasm	599	0.53	2.982E-32
GOTERM_MF_5	GO:0032559 ~ adenyl ribonucleotide binding	892	0.78	1.822E-20
GOTERM_MF_5	GO:0004672 ~ protein kinase activity	387	0.34	1.894E-13
GOTERM_MF_5	GO:0000287 ~ magnesium ion binding	294	0.26	2.071E-11
GOTERM_MF_5	GO:0016462 ~ pyrophosphatase activity	448	0.39	1.642E-08
GOTERM_MF_5	GO:0035257 ~ nuclear hormone receptor binding	64	0.06	1.128E-07
GOTERM_MF_5	GO:0003713 ~ transcription coactivator activity	147	0.13	1.259E-07
GOTERM_MF_5	GO:0004527 ~ exonuclease activity	45	0.04	1.407E-05
GOTERM_MF_5	GO:0019787 ~ small conjugating protein ligase activity	108	0.09	0.0010606
GOTERM_MF_5	GO:0019901 ~ protein kinase binding	95	0.08	0.0050729
GOTERM_MF_5	GO:0050136 ~ NADH dehydrogenase (quinone) activity	34	0.03	0.0085249

**Table 5:** GO annotation of identified proteins

Category	Term	Count	%	Benjamini
GOTERM_BP_5	GO:0002526 ~ acute inflammatory response	28	0.47	1.7E-17
GOTERM_BP_5	GO:0006956 ~ complement activation	15	0.25	1.06E-09
GOTERM_BP_5	GO:0016485 ~ protein processing	20	0.33	1.93E-08
GOTERM_BP_5	GO:0030193 ~ regulation of blood coagulation	13	0.22	2.19E-08
GOTERM_BP_5	GO:0006958 ~ complement activation, classical pathway	12	0.20	2.2E-08
GOTERM_BP_5	GO:0030195 ~ negative regulation of blood coagulation	11	0.18	2.31E-08
GOTERM_BP_5	GO:0051604 ~ protein maturation	21	0.35	2.32E-08
GOTERM_BP_5	GO:0050819 ~ negative regulation of coagulation	11	0.18	8.73E-08
GOTERM_BP_5	GO:0019724 ~ B cell mediated immunity	14	0.23	2.36E-07
GOTERM_BP_5	GO:0002253 ~ activation of immune response	16	0.27	2.8E-06
GOTERM_CC_5	GO:0044444 ~ cytoplasmic part	190	3.16	4.07E-16
GOTERM_CC_5	GO:0005737 ~ cytoplasm	242	4.03	1.58E-14
GOTERM_CC_5	GO:0060205 ~ cytoplasmic membrane-bounded vesicle lumen	17	0.28	8.44E-14
GOTERM_CC_5	GO:0031093 ~ platelet alpha granule lumen	16	0.27	4.9E-13
GOTERM_CC_5	GO:0016023 ~ cytoplasmic membrane-bounded vesicle	42	0.70	1.43E-09
GOTERM_CC_5	GO:0031410 ~ cytoplasmic vesicle	44	0.73	1.16E-08
GOTERM_CC_5	GO:0030141 ~ secretory granule	22	0.37	3.97E-08
GOTERM_CC_5	GO:0048770 ~ pigment granule	16	0.27	5.49E-08
GOTERM_CC_5	GO:0000323 ~ lytic vacuole	23	0.38	9.38E-08
GOTERM_CC_5	GO:0005788 ~ endoplasmic reticulum lumen	15	0.25	1.04E-07
GOTERM_MF_5	GO:0004867 ~ serine-type endopeptidase inhibitor activity	19	0.32	5.63E-09
GOTERM_MF_5	GO:0004252 ~ serine-type endopeptidase activity	20	0.33	2.61E-06
GOTERM_MF_5	GO:0008236 ~ serine-type peptidase activity	21	0.35	3.68E-06
GOTERM_MF_5	GO:0004175 ~ endopeptidase activity	31	0.52	3.8E-06
GOTERM_MF_5	GO:0008201 ~ heparin binding	15	0.25	2.18E-05
GOTERM_MF_5	GO:0005509 ~ calcium ion binding	51	0.85	2.98E-05
GOTERM_MF_5	GO:0004869 ~ cysteine-type endopeptidase inhibitor activity	7	0.12	0.010385
GOTERM_MF_5	GO:0051920 ~ peroxiredoxin activity	4	0.07	0.02113

### Tissues-specific analysis of mRNAs and proteins

All the known mRNAs and proteins were performed tissues-specific analysis. The results of mRNA analysis showed 8,605 of 13,895 genes were associated with 100 tissues, and were significantly correlated (Benjamini < 0.05) with 50 tissues. According to gene number, the top 5 ranking tissues were brain (3,987 genes), placenta (1,872 genes), epithelium (1,595 genes), lung (1,426 genes) and liver (1,110 genes) (Table 6 and Additional file 10). However, all the proteins were correlated with 33 tissues, and significantly correlated (Benjamini < 0.05) with only 19 tissues, including the components closely relative tissues of milk, such as plasma, blood, milk and mammary gland. More interestingly, the top five enriched tissues were liver (138 proteins), placenta (128 proteins), skin (75 proteins), lung (74 proteins) and plasma (73 proteins), and the highly correlated tissues were plasma, liver and milk (Table 7 and Additional file 8). These results suggest that mRNAs and proteins in porcine milk exosomes may have originated from multiple tissues.

**Table 6:** mRNAs expressed in a tissue-specific manner

Category	Term	Count	%	Benjamini
UP_TISSUE	Epithelium	1,595	1.40	1.77E-68
UP_TISSUE	Placenta	1,872	1.65	2.06E-44
UP_TISSUE	Skin	1,079	0.95	1.66E-38
UP_TISSUE	Uterus	1,021	0.90	4.31E-35
UP_TISSUE	Brain	3,987	3.51	5.68E-34
UP_TISSUE	Lung	1,426	1.25	1.43E-28
UP_TISSUE	Cervix carcinoma	255	0.22	3.19E-19
UP_TISSUE	Muscle	492	0.43	7.94E-18
UP_TISSUE	Liver	1,110	0.98	9.72E-16
UP_TISSUE	Lymph	400	0.35	1.43E-13
UP_TISSUE	Fetal brain cortex	153	0.13	2.22E-13
UP_TISSUE	Eye	582	0.51	4.51E-13
UP_TISSUE	Platelet	338	0.30	4.58E-13
UP_TISSUE	Bone marrow	431	0.38	4.91E-13
UP_TISSUE	Cervix	308	0.27	7.3E-13
UP_TISSUE	Cajal-Retzius cell	137	0.12	3.21E-11
UP_TISSUE	Colon	642	0.56	1.35E-10
UP_TISSUE	Urinary bladder	137	0.12	1.06E-09
UP_TISSUE	B-cell lymphoma	93	0.08	7.21E-09
UP_TISSUE	Colon carcinoma	124	0.11	7.39E-09
UP_TISSUE	T-cell	199	0.17	2.83E-08
UP_TISSUE	Umbilical cord blood	197	0.17	5.14E-08
UP_TISSUE	Mammary gland	240	0.21	6.05E-08
UP_TISSUE	Ovary	452	0.40	4.35E-07
UP_TISSUE	Kidney	765	0.67	1.81E-06
UP_TISSUE	Pancreas	519	0.46	4.43E-06
UP_TISSUE	B-cell	160	0.14	6.34E-05
UP_TISSUE	Teratocarcinoma	297	0.26	0.000105
UP_TISSUE	Adipose tissue	108	0.09	0.000142
UP_TISSUE	Human small intestine	51	0.04	0.001389
UP_TISSUE	Melanoma	176	0.15	0.001846
UP_TISSUE	Leukemia	46	0.04	0.001979
UP_TISSUE	Keratinocyte	80	0.07	0.003529
UP_TISSUE	Ovarian carcinoma	90	0.08	0.00707
UP_TISSUE	Fetal kidney	106	0.09	0.007677
UP_TISSUE	Renal cell carcinoma	42	0.04	0.008965
UP_TISSUE	Umbilical vein	27	0.02	0.008996
UP_TISSUE	Pituitary	86	0.08	0.010397
UP_TISSUE	Bone	42	0.04	0.014
UP_TISSUE	Skeletal muscle	295	0.26	0.014765
UP_TISSUE	Endometrium carcinoma cell line	19	0.02	0.020668
UP_TISSUE	Embryonic kidney	48	0.04	0.021149
UP_TISSUE	Bladder	48	0.04	0.021149
UP_TISSUE	Hepatoma	128	0.11	0.021469
UP_TISSUE	Embryo	183	0.16	0.026251
UP_TISSUE	Mammary carcinoma	59	0.05	0.033882
UP_TISSUE	Breast	53	0.05	0.036028
UP_TISSUE	Hypothalamus	73	0.06	0.036155
UP_TISSUE	Dendritic cell	49	0.04	0.041269
UP_TISSUE	Fetal brain	391	0.34	0.041334
UP_TISSUE	Carcinoma	33	0.03	0.050043

**Table 7:** Proteins expressed in a tissue-specific manner

Category	Term	Count	%	Benjamini
UP_TISSUE	Plasma	73	1.22	3.36E-52
UP_TISSUE	Liver	138	2.30	3.01E-31
UP_TISSUE	Milk	17	0.28	2.57E-16
UP_TISSUE	Fetal brain cortex	32	0.53	6.22E-15
UP_TISSUE	Cajal-Retzius cell	30	0.50	2.98E-14
UP_TISSUE	Bile	11	0.18	6.73E-10
UP_TISSUE	Placenta	128	2.13	8.25E-09
UP_TISSUE	Urine	10	0.17	1.45E-07
UP_TISSUE	Saliva	12	0.20	6.37E-07
UP_TISSUE	Skin	75	1.25	5.48E-06
UP_TISSUE	Platelet	34	0.57	7.73E-06
UP_TISSUE	Neutrophil	7	0.12	0.000105
UP_TISSUE	Pancreas	44	0.73	0.000382
UP_TISSUE	Mammary gland	24	0.40	0.000715
UP_TISSUE	B-cell lymphoma	12	0.20	0.002124
UP_TISSUE	Blood	29	0.48	0.021296
UP_TISSUE	Adipose tissue	12	0.20	0.024482
UP_TISSUE	Colon	43	0.72	0.025496
UP_TISSUE	Ovary	32	0.53	0.047235

#### KEGG pathway analysis of mRNAs and proteins

**Fig. 6:**
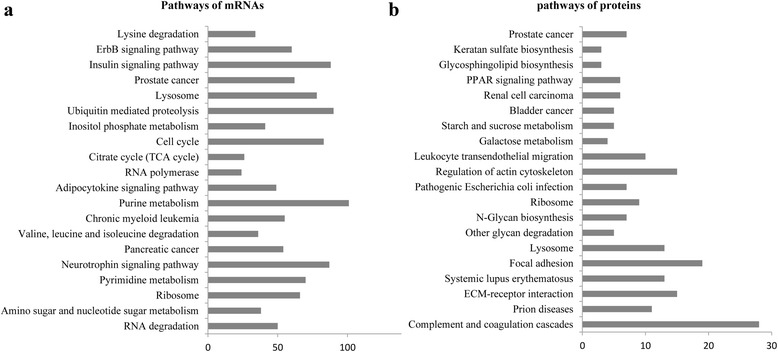
KEGG pathway analysis. **a** Pathway analysis of mRNAs. **b** Pathway analysis of proteins

Due to the incomplete porcine bioinformatics resources in software DAVID [30], we selected the human database as reference. For mRNA, only 8,605 of 13,895 genes were enriched in 63 KEGG pathways, and the top 20 pathways were involved in various substance metabolisms, degradation, signaling pathway and some diseases pathways. Interestingly, we got 83 genes in cell cycle pathways (Fig. 6a and Additional file 10). For proteins, the results showed that only 426 of the 571 proteins (known proteins) were found to be enriched using KEGG pathway in the database. These 426 proteins were enriched in 20 pathways (Fig. 6b and Additional file 8), most associated with immunity and diseases.

## Discussions

In the present study, we totally obtained 13,895 known genes and 2,409 putative novel genes in porcine milk exosomes. It was reported 10,948 mRNA transcripts in rats whey [21] and 19,320 transcripts in bovine milk whey exosome by mRNA microarray. Moreover, in human milk, 14,070 transcripts were found in fat globules [37]. Some of milk protein genes (CSN2, CSN3 and CSN1S1), ribosome-related proteins genes (RPS18, RPL18 and RPLP1) and other genes (e.g UBA52, FABP3 and EEF1A1) were highly expressed in the previous researches [21, 37], which were in accordance with this study (Additional file 3). Furthermore, some genes such as LALBA, TPT1, SPP1 and FASN were not found in rats whey [21], bovine milk whey exosome and human milk fat globules [37]. Additionally, the randomly selected 14 mRNAs among top 50 were further confirmed using qRT-PCR. Differences of mRNAs in milk or milk exosome exist among species, possibly indicating different functions of milk among species.

One of the aims in the present study was to explore the protein content of porcine milk exosomes using proteomics. In the present study, we observed 639 proteins (Fig. 1b) including the exosomal marker proteins CD9 [38] and CD63 [39] using Western blotting (Fig. 1a), as well as the heat shock protein family members HSPA 90 B1, HSPA13, HSPA5, HSPA 9, HSPB1 and HSPCB (Additional file 4), which have been reported in previous exosome research [7, 8, 16]. The Western blotting results in our study confirmed that we successfully isolated porcine milk exosomes. Previous study using iTRAQ identified 2,971 milk proteins with 2,350 from exosomes, 1,012 from MFGM, and 748 from whey (FDR = 0.1%) [30], another study found 2,107 proteins in bovine milk exosomes, including the major exosomal marker proteins lactadherin/MFGE8 and TSG 101 (FDR = 0.05% for proteins and 0.2% for peptides) [29], the actin family members ACTC1, ACTN1, ACTN2, and ACTN4 are cell-specific proteins likely involved in exosome biogenesis and potentially other exosome functions [7], which were also present in porcine milk exosomes. However, xanthine oxidase (~147 kDa), Butyrophilin (~59 kDa), lactadherin/MGF8 (~47 kDa) and adipophilin/perilipin-2 (~49 kDa) and MFGM were identified in bovine milk exosomes [29, 40], which were not detected in porcine milk exosomes. It has also been suggested that exosomes from different sources might contain different components [16] and may play tissue-specific roles in intracellular communication and immune function [41–43].

In COG ontology analysis of mRNAs and proteins, we obtained 10 conserved proteins and 18 mRNAs relative to cell cycle. Additionally, many genes and proteins involved in cell cycle and immunity related pathways by KEGG pathways analysis. Then, we randomly selected 10 proteins for Western blotting analysis. Platelet-derived growth factor (PDGF) acts as a potential binding pattern mitogen for mesenchymal cells both in vitro and in vivo [44]. Epidermal growth factor (EGF) plays an important role in regulating cell proliferation and differentiation during development [45]. Thrombospondin1 (THBS1), cysteine-rich protein 61 (Cyr61) and connective tissue growth factor (CTGF) were all involved in the transforming growth factor-beta (TGF-β) signaling pathway [46]. High-temperature requirement A3 (HtrA3) inhibits BMP-4, BMP-2 and TGF-β1 signaling [47]. Lactoferrin (LTF) functions in inflammation [48]. Myostatin (MSTN) was a negative regulator of myogenesis and has been implicated in the regulation of adiposity and controlling the structure and function of tendons [49]. IGFBP-7 acts through autocrine/paracrine pathways to inhibit BRAF-MEK-ERK signaling and induces senescence and apoptosis in cells containing the BRAF oncogene [50]. Additionally, IGFBP-7 inhibits cell growth and induces apoptosis in RKO and SW620 cells [51]. Confirmation of the presence of these 10 proteins in porcine milk exosomes suggests a possible function in the regulation of immunity, cell proliferation and possibly other pathways.

All previously reported exosomal proteins were cytosolic, and many of them were associated with the plasma membrane or membranes of endocytic compartments [7]. Most of the genes and proteins identified in the present study were relatived to cytoplasm or cytoplasmic GO terms (Tables 4 and 5). Analysis of GO, KEGG and COG annotations suggested that most porcine milk exosome genes and proteins might function in activation, immunity and cell cycle. KEGG analysis revealed that four pathways (ECM-receptor interaction, Focal adhesion, Regulation of actin cytoskeleton and Leukocyte transendothelial migration) were enriched in both bovine [29] and porcine milk exosomes (Fig. 6b, Additional file 8). Above results indicate a similar function in different species. Additionally, recent reports showed that the bovine milk exosomes were able to exert endocytosis and transferred their contained molecules to other cells [52]. In this study, proteins in porcine milk exosome were predicated to be involved in pathways of starch and sucrose metabolism, other glycan degradation, N-Glycan biosynthesis, galactose metabolism and glycosphingolipid biosynthesis (Fig. 6b, Additional file 8), it was deduced that porcine milk exosomes might transfer encapsulated materials, which could mediated by those proteins and played key roles in different physiological and pathological conditions. Meanwhile, the KEGG analysis of mRNAs showed lots of genes enriched in Purine metabolism, Pyrimidine metabolism, Insulin signaling pathway, Cell cycle and RNA degradation pathways, which were different to predicated pathways in the KEGG analysis of porcine milk exosomes proteins.

It is reported the viral RNA (hepatitis C virus) was able to transfer to infected cells (plasmacytoid dendritic cells) and trigger an innate immune response, depending on membrane vesicle trafficking [53]. The glioblastoma cells derived-exosome could deliver a specific mRNA transcript to endothelia cells followed by generating functional proteins for patients [54]. When incubated with NIH-3 T3 cells, milk-derived microvesicles could transfer bovine milk related transcripts to living cells and affect the calf’s gastrointestinal development and immune systems [6]. Additionally, recent reports showed that bovine milk exosomes can be uptaken by endocytosis, depending on cell exosome surface glycoproteins [52].The uptaking exosome further affected gene expression [55]. And exosome can also be incorporated into differentiated human cells with containing RNA. These data collectively indicate the exosomes could not only deliver the encapsulated miRNAs, mRNAs and proteins to recipient cells, but also make their specific functions on immunity, thereafter play a key role in different physiological and pathological conditions. Our results provided extensively mRNAs and proteins data, which are beneficial to understand how milk regulates health and development of newborns by exosomes.

## Conclusions

In this study, we identified 16,304 mRNAs and 639 proteins in porcine milk exosomes by RNA-sequencing and proteomic analysis, and many of mRNAs and proteins were predicted to be involved in immunity, proliferation and cellular signaling, which would be closely associated with piglets development and healthy. These findings provided a large amount of informations and contributed to increased understanding of the role of genes and proteins in milk exosomes, and build a foundation for future studies on their physiological functions and regulatory mechanisms.

## Additional files

Additional file 1: mRNA expression. (XLSX 1186 kb)

Additional file 2: Novel transcripts. (XLSX 184 kb)

Additional file 3: Different top 50 mRNAs expression. (XLSX 20 kb)

Additional file 4: The 639 identified proteins analysis. (XLSX 400 kb)

Additional file 5: Figure S1. Peptide length distribution of identified proteins. a, b, c, d, and e represent the peptide length distribution of 10, 13, 6, 8, and *Sus_Scrofa* proteins, respectively. (TIFF 1171 kb)

Additional file 6: Figure S2. Peptide and spectrogram distribution of identified proteins. a, b, c, d, and e represent the peptide and spectrogram distribution of 10, 13, 6, 8, and *Sus_Scrofa* proteins, respectively. (TIFF 942 kb)

Additional file 7: Figure S3. Distribution of protein sequences coverage. a, b, c, d, and e represent 10, 13, 6, 8, and *Sus_Scrofa,* respectively. (TIFF 1199 kb)

Additional file 8: Proteins bioinformatics analysis. (XLSX 28 kb)

Additional file 9: Figure S4. COG annotation of identified proteins. a, b, c, and d represent 10, 13, 6, and 8, respectively. (TIFF 1251 kb)

Additional file 10 mRNAs bioinformatics analysis.xlsx. (XLSX 218 kb)

## Abbreviations

ACN: Acetonitrile
ACTC1: Alpha-cardiac actin one
ACTN2: Alpha-actin two
ACTN4: Alpha-actin four
AGC: Automatic gain control
BCA: Bicinchoninic acid
BMP-2: Bone morphogenetic protein 2
BMP-4: Bone morphogenetic protein 4
BP: Biological Process
CC: Cellular Component
CD24: Tetraspan-integrin complexes 24
CD63: Tetraspan-integrin complexes 63
CD9: Tetraspan-integrin complexes 24
COG: Cluster of Orthologous Groups
CSN1S1: Casein alpha s1
CSN2: Beta-casein
CSN3: κ-casein
CTGF: Connective tissue growth factor
Cyr61: Cysteine-rich protein 61
DTT: DL-Dithiothreitol
EEF1A1: Elongation factor 1 alpha-1
EGF: Epidermal growth factor
FABP3: Fatty-acid binding protein 3
FASN: Fatty acid synthase
FDR: False discovery rate
GO: Gene Ontology
HCD: High-energy collision dissociation
HRP: Horseradish Peroxidase
Hsp70: 70-kDa heat-shock protein
HSPA 9: Heat shock 70kD protein 9
HSPA 90 B1: heat shock protein 90 kDa beta,member 1
HSPA13: Heat shock protein 70 kDa family, member 13
HSPA5: Heat shock 70 kDa protein 5
HSPB1: Heat shock 27 kDa protein 1
HSPCB: Heat shock 90kD protein beta
HTRA3: High-temperature requirement A3
IGFBP-7: Insulin-like growth factor binding protein 7
iTRAQ: Isobaric tag for relative and absolute quantitation
KEGG: Kyoto Encyclopedia of Genes and Genomes
LALBA: Lactalbumin alpha
LC-MS/MS: Liquid chromatography with tandem mass spectrometric
LTF: Lactoferrin
MF: Molecular Function
MFG: Milk fat globules
MFGE8: Milk fat globule-EGF factor 8
MFGM: Milk fat globule membrane
MGF: Mammary gland factor
MHC-class II: Major histocompatibility complex class II
miRNAs: MicroRNAs
mRNAs: Messager RNA
MSTN: Myostatin
NADH: Nicotinamide adenine dinucleotide
ncRNA: Noncoding RNA
PDGF: Platelet-derived growth factor
PDGFA: Platelet-derived growth factor A chain
PMSF: Phenylmethanesulfonyl fluoride
QC: Quality control
RIPA: Radio-immunoprecipitation assay
RNA-Seq: RNA-sequencing
RPL18: 60S ribosomal subunit protein L18
RPLP1: Ribosomal phosphoprotein P1
RPS18: Ribosomal protein S18
rRNA: Ribosomal RNA
SPP1: Secreted phosphoprotein 1
TGFB-3: Transforming growth factor-beta 3
TGF-β1: Transforming growth factorβ1
THBS1: Thrombospondin1
TPT1: Translationally controlled tumor protein 1
TSG 101: Tumor susceptibility gene 101
UBA52: Ubiquitin A-52 ribosomal protein
