# Supplementary figures and images for: Revelation of mRNAs and proteins in porcine milk exosomes by transcriptomic and proteomic analysis
Source: BMC Vet Res. 2017 Apr 13;13:101. doi: 10.1186/s12917-017-1021-8 (PMC5390444; doi:10.1186/s12917-017-1021-8)

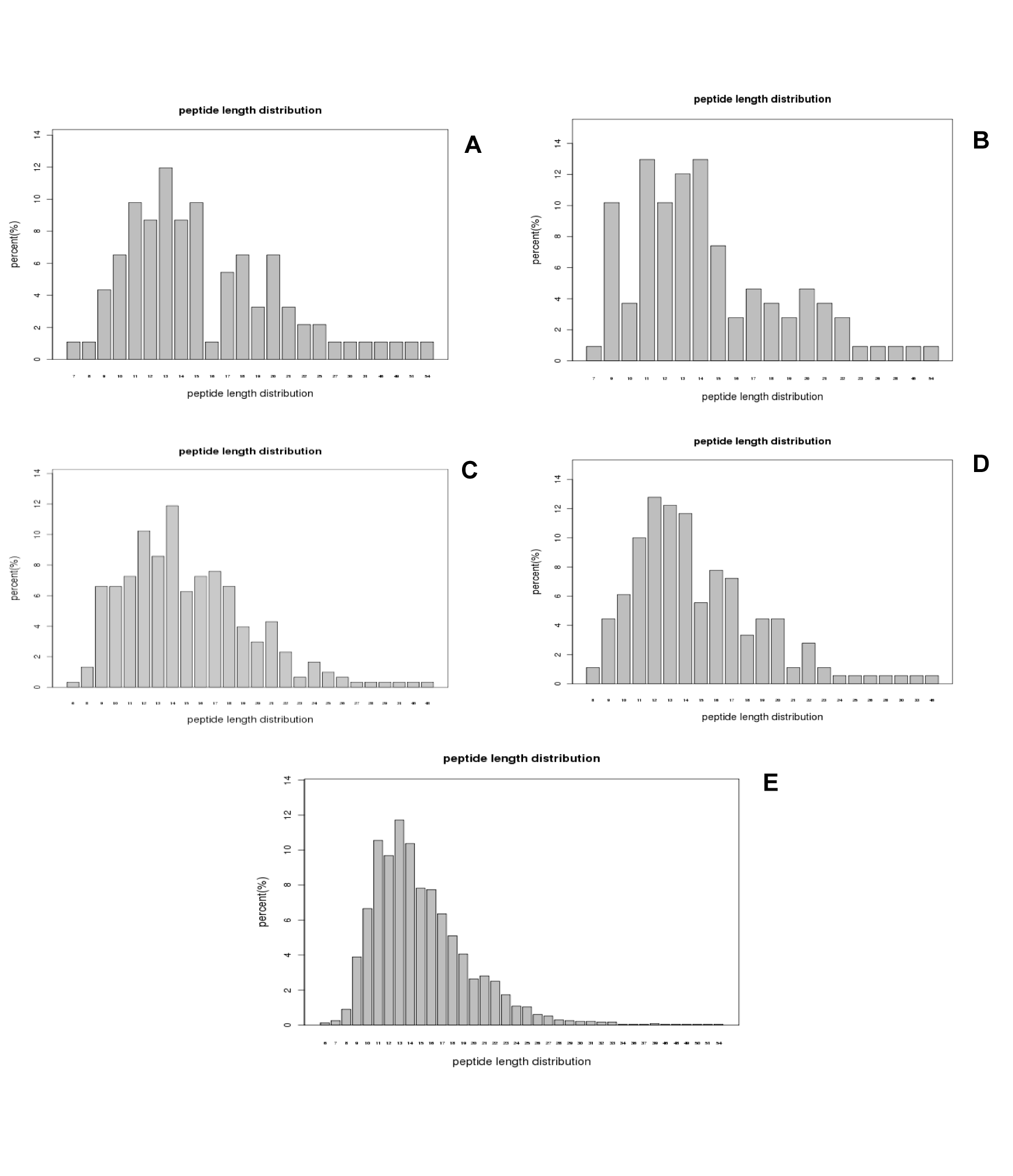

Supplement: Supplementary file 5 — Peptide length distribution of identified proteins. a, b, c, d, and e represent the peptide length distribution of 10, 13, 6, 8, and Sus_Scrofa proteins, respectively. (TIFF 1171 kb) [file 12917_2017_1021_MOESM5_ESM.tif]

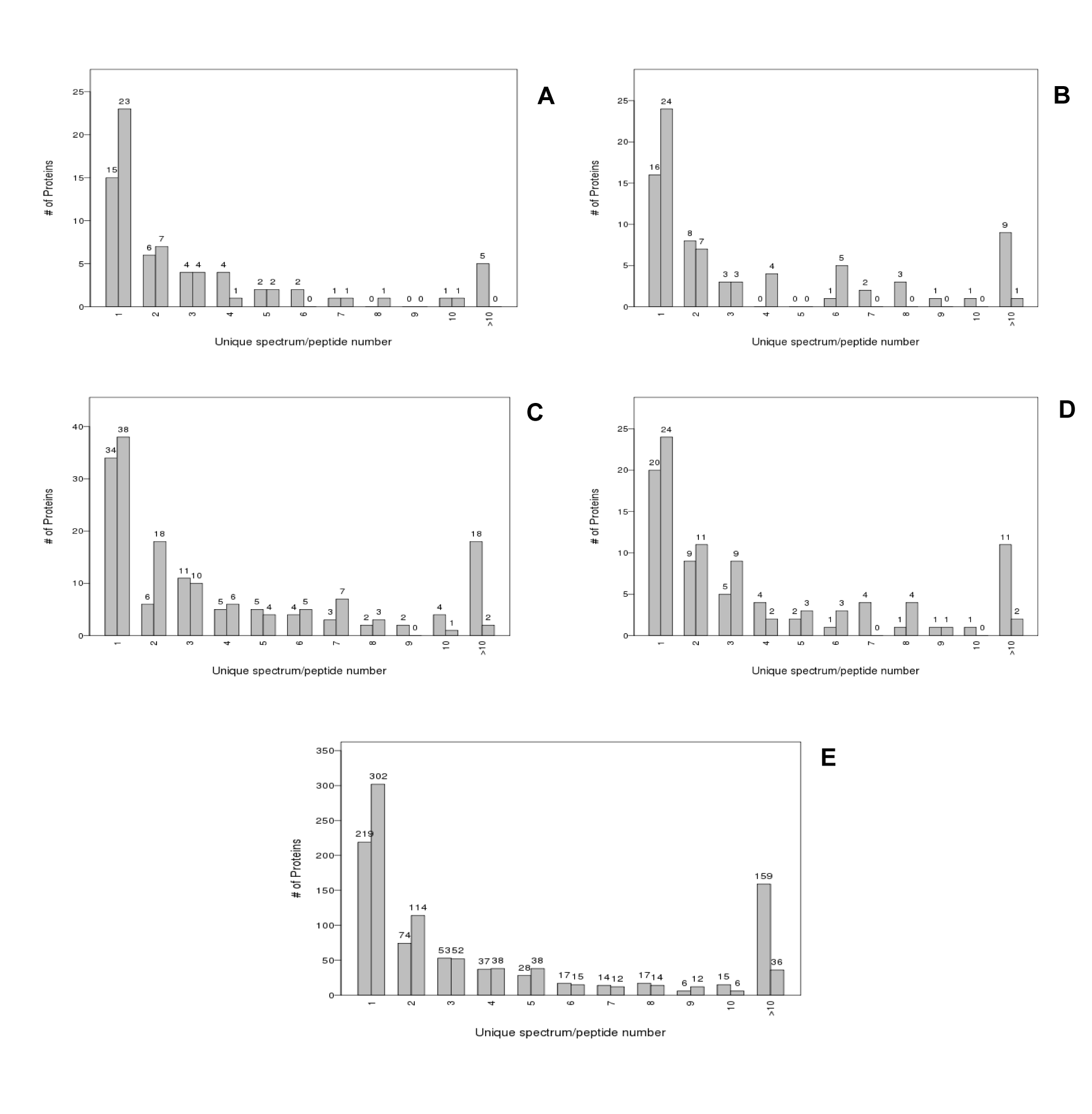

Supplement: Supplementary file 6 — Peptide and spectrogram distribution of identified proteins. a, b, c, d, and e represent the peptide and spectrogram distribution of 10, 13, 6, 8, and Sus_Scrofa proteins, respectively. (TIFF 942 kb) [file 12917_2017_1021_MOESM6_ESM.tif]

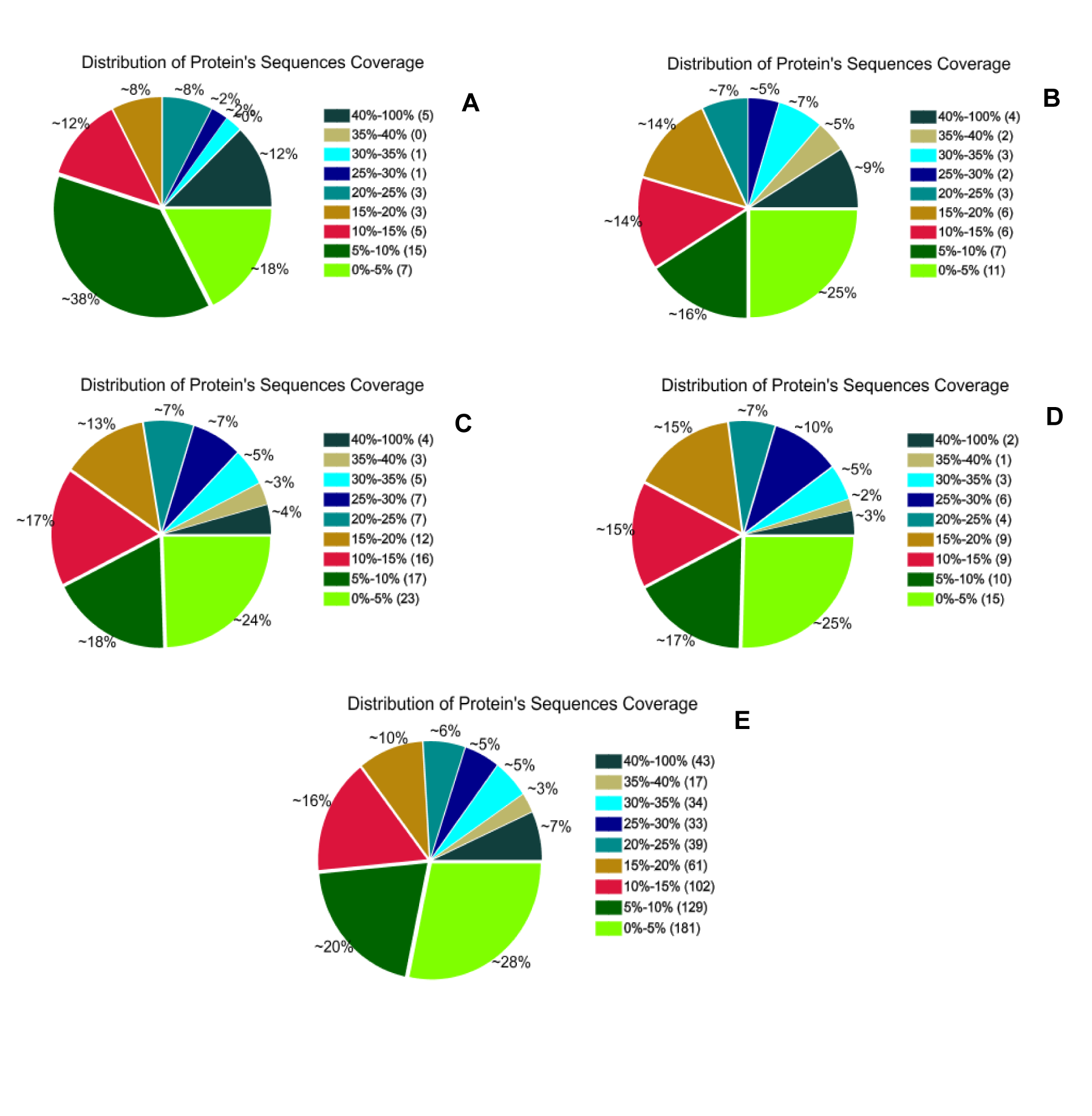

Supplement: Supplementary file 7 — Distribution of protein sequences coverage. a, b, c, d, and e represent 10, 13, 6, 8, and Sus_Scrofa, respectively. (TIFF 1199 kb) [file 12917_2017_1021_MOESM7_ESM.tif]

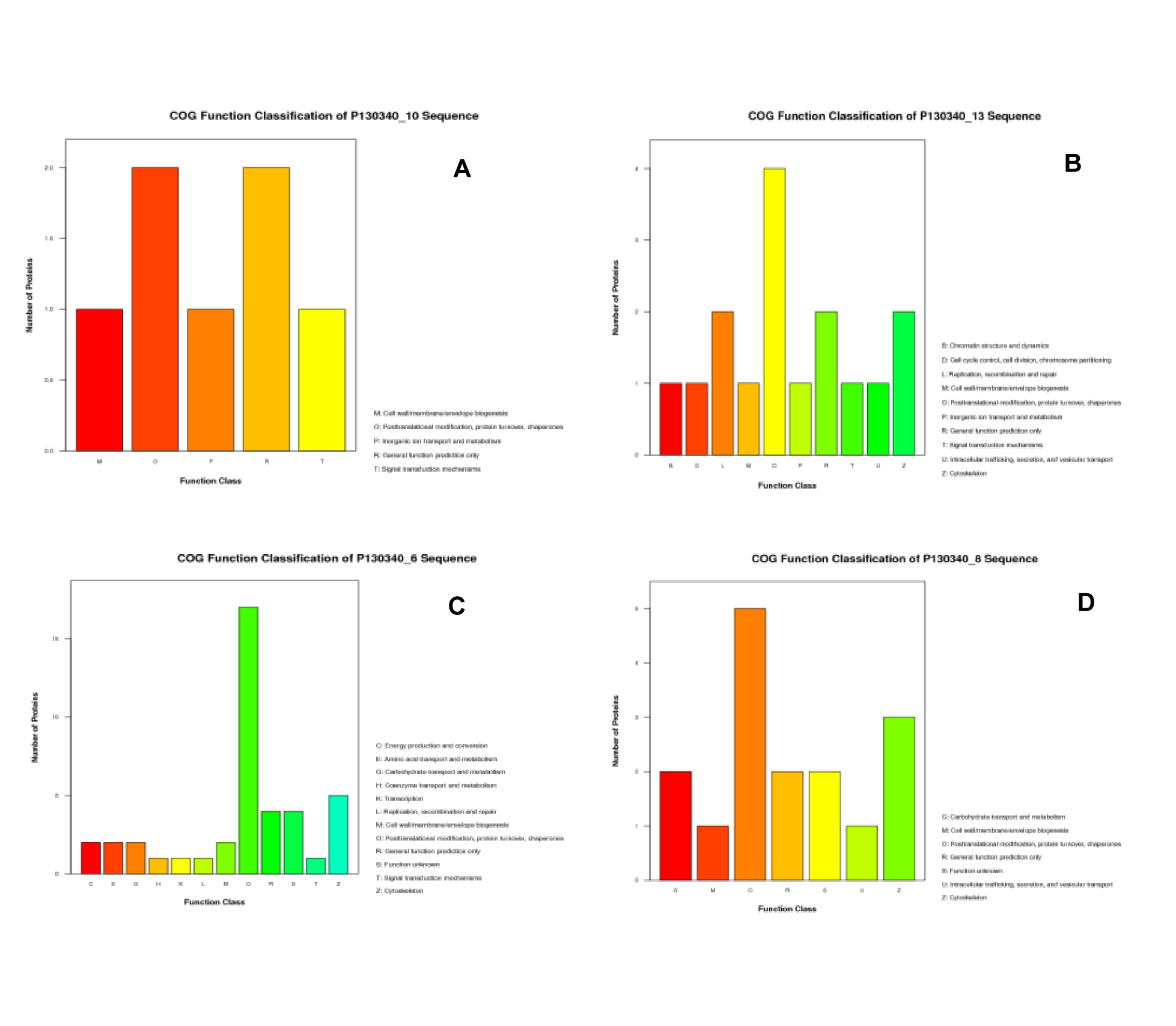

Supplement: Supplementary file 9 — COG annotation of identified proteins. a, b, c, and d represent 10, 13, 6, and 8, respectively. (TIFF 1251 kb) [file 12917_2017_1021_MOESM9_ESM.tif]
